# High gamma EEG responses to emotional stimuli in virtual reality: insights from local activation and distributed characteristics

**DOI:** 10.3389/fnhum.2025.1623331

**Published:** 2025-07-23

**Authors:** Shasha Xiao, Nadia Youssef, Qingxun Zhang, Xiaoqian Lin, Ziquan Qiu, Wenjie Liu, Xianglian Meng, Minchang Yu

**Affiliations:** ^1^School of Computer Science and Information Engineering, Changzhou Institute of Technology, Changzhou, China; ^2^Knowlepsy, Marseille, Provence-Alpes-Côte d'Azur, France

**Keywords:** electroencephalogram, virtual reality, emotion classification, high gamma oscillations, spectral power, brain networks

## Abstract

**Introduction:**

High frequency electroencephalogram (EEG) activity, particularly in the high gamma range, plays an important role in research on human emotions. However, the current understanding of high gamma EEG responses to emotional stimuli in virtual reality (VR) remains limited, especially regarding local activations and distributed network characteristics during different emotional states.

**Methods:**

In this study, EEG responses to positive and negative VR stimuli were analyzed. EEG data were recorded from 19 participants as they viewed 4-second VR videos designed to elicit positive and negative responses. Two neural signatures were examined: high gamma band (53–80 Hz) spectral power and brain network features (nodal/local efficiency).

**Results and discussion:**

Spectral power analysis revealed valence-specific spatial patterns in spectral power, with significantly higher frontal gamma activity during positive states and increased right temporal gamma power during negative states. Network analysis revealed elevated local efficiency during positive emotions, indicating enhanced modular connectivity. Machine learning classification demonstrated higher accuracy for spectral power features (73.57% ± 2.30%) compared to nodal efficiency (69.51% ± 2.62%) and local efficiency (65.03% ± 1.33%), with key discriminators identified in frontal, temporal, and occipital regions. These findings suggest that localized high gamma activity provides more direct discriminative information for emotion recognition in VR than network topology metrics, advancing the understanding of neurophysiological responses in immersive VR environments.

## 1 Introduction

Emotions represent complex psychological and physiological states that motivate and organize cognition and action to facilitate adaptive responses to environmental challenges (Panksepp, 2004; Mauss et al., 2005). Identifying robust neural biomarkers for emotions is crucial for understanding the cognitive and neural mechanisms of emotion processing. It also has significant implications for clinical applications (e.g., early diagnosis of affective disorders) and advancements in brain-computer interfaces (Zhang J. et al., 2024). While traditional neuroimaging modalities like functional magnetic resonance imaging (fMRI) provide high spatial resolution, electroencephalogram (EEG) demonstrates a higher temporal resolution, enabling accurate detection and recording of the temporal dynamics of brain activity (Yang et al., 2020; Maithri et al., 2022).

Recent research has increasingly focused on the gamma band (30–80 Hz), particularly the high gamma sub-band (typically >50 Hz) as it is a critical neural signature of emotion processing. High gamma oscillations are spatially localized neurophysiological signals that reflect synchronized firing of local neuronal assemblies. They have been closely linked to localized cortical computation during sensory processing, emotional processing (Yang et al., 2020), memory consolidation (Kanta et al., 2019), and cognitive function (Corlier et al., 2016). In the context of affective neuroscience, a growing body of research indicates that high-gamma power is highly sensitive to emotional stimuli and can differentiate between affective states. Most of these studies have reported enhanced responses to emotional stimuli compared to neutral stimuli (Onton and Makeig, 2009; Martini et al., 2012; Boucher et al., 2015; Yang et al., 2020). For instance, intracranial recordings demonstrated increased high gamma power for unpleasant pictures compared to pleasant ones, particularly in bilateral occipitotemporal visual areas (200–1,000 ms) and a subsequent decrease in lateral prefrontal cortex activity (500–800 ms) (Boucher et al., 2015). Notably, high gamma activity also appears to offer advantages for emotion decoding over traditional lower frequency bands. Yang et al. (2020) directly compared multiple EEG frequency bands and showed that features from the high gamma band yielded superior classification accuracy for emotional states relative to alpha, beta, and theta bands. Overall, high gamma oscillations present a promising neural marker for emotion-related processing.

Consistently, studies have shown that emotional stimuli—especially those with negative valence—often evoke enhanced high gamma responses (Güntekin and Basar, 2007; Onton and Makeig, 2009; Martini et al., 2012; Boucher et al., 2015). These oscillations are hypothesized to reflect rapid integration of sensory-affective inputs across key regions, including the prefrontal cortex, occipital cortex, medial temporal cortex, and limbic regions such as the anterior cingulate cortex and insula (Ochsner et al., 2009, 2012).

Spectral power characteristics have been widely utilized in EEG-based emotion recognition due to their capability to detect localized neural synchronization (Liu et al., 2025). For instance, Miranda-Correa et al. evaluated EEG-based emotion recognition on the AMIGOS dataset using spectral power features, reporting F1-scores of 0.576 for valence and 0.592 for arousal classification with a Gaussian Naive Bayes classifier in short-video experiments (Miranda-Correa et al., 2018). Koelstra et al. extracted power spectral density (PSD) features from 32 EEG electrodes, achieving accuracies of 62.0 and 57.6% during the two-class classification of arousal and valence using a user-independent model (Koelstra et al., 2011). These findings emphasize the diagnostic significance of regional activation patterns but do not completely encompass the distributed network properties that support emotional processing—an important limitation in light of the brain’s modular organization. In other words, the cognitive process could not only be analyzed using the power distribution across different brain regions, but the study of interactions between brain areas from a brain network perspective may reveal potential network features that could be used for emotion recognition.

To address this gap, graph theory-based network analysis offers a complementary framework for quantifying global integration and local segregation of functional connectivity (Kumar et al., 2024; Xu et al., 2024). In recent years, graph theory-based approaches to analyzing brain networks have emerged as a prominent framework in neuroscience research. This methodology conceptualizes the brain as a complex network composed of interconnected nodes and functional modules. By quantifying the connectivity patterns between these nodes, researchers can uncover the organizational principles underlying information processing and emotion regulation in the brain. Prior studies have demonstrated the potential of graph theoretical metrics (e.g., global/local efficiency) in emotion classification (Bullmore and Sporns, 2009). For instance, Kılıç and Aydın classified contrasting discrete emotional states (e.g., happiness vs. sadness, calmness vs. excitement) using EEG-based graph theoretical network measures (Kılıç and Aydın, 2022). They employed Pearson correlation (PC) and Spearman correlation (SC) to estimate functional connectivity from EEG segments of 6 and 12 s. Five graph theoretical measures – the clustering coefficient (CC), transitivity (T), global efficiency (GE), local efficiency (LE), and modularity (Q) – were extracted as features. Using support vector machines (SVMs), they achieved classification accuracies ranging from 67.31 to 73.91% for specific emotion pairs. Notably, integration measures (GE, LE) outperformed segregation measures (CC, T, Q) in emotion classification, with the highest accuracy of 80.65% for distinguishing excitement from calmness. These findings highlight the utility of graph theoretical measures in capturing the neurofunctional dynamics of emotional states. While both spectral power and graph theoretical approaches yield comparable classification accuracies in isolated implementations, direct comparisons of their discriminative power remain unknown.

It is noteworthy that, in the majority of current studies, emotions were elicited within two-dimensional environments, particularly in laboratory settings that employ mood induction procedures (MIPs) using stimuli such as images and videos. However, this common paradigm of emotion induction, based primarily on laboratory contexts, differs distinguishably from real-world settings and can not fully capture the psychological and physiological responses experienced in real-world conditions (Zhang C. et al., 2024). Compared to 2D stimuli, virtual reality (VR) stimuli provide a highly immersive and realistic virtual setting that allows for greater experimental control over emotional induction paradigms, resulting in more authentic emotional responses. The study by Chirico and Gaggioli also provided evidence that VR stimuli can elicit a variety of emotions in a manner similar to real experiences (Chirico and Gaggioli, 2019). Consequently, the use of VR is considered a potential tool for capturing and differentiating brain patterns during various emotional states under more realistic conditions, by bridging the gap between laboratory and real world environments (Choi et al., 2023; Zhang C. et al., 2024). In Tian et al.’s study comparing emotion-related EEG responses in 2D and 3D environments, significantly greater emotional arousal was observed in the 3D setting compared to the 2D setting (Tian et al., 2021). Similarly, He et al. discovered that head-mounted VR environments led participants to perceive more realism than 2D environments (He et al., 2018). Given the aforementioned discrepancies between 2D displays and real-world applications, there may be differences in EEG dynamics between 3D and 2D presentations (Kakkos et al., 2019; Manshouri et al., 2020; Yu et al., 2021). Therefore, using VR MPSs to simulate real-world conditions in a controlled laboratory setting is advisable.

Our lab previously introduced the VREED dataset and conducted preliminary analyses using traditional EEG frequency bands (e.g., theta, alpha, beta, and low-gamma) to classify emotional states in immersive VR environments (Yu et al., 2022). However, the potential of the high-gamma band and graph-theoretical metrics remained unexplored. The present study directly builds on this earlier work by leveraging the same VR-evoked EEG dataset and extending the analysis to these underexplored high-gamma features and brain network topological properties, providing deeper insight into emotion-specific neural dynamics under VR conditions.

Despite these advances, current understanding of emotion-related neural mechanisms in VR remains limited, particularly regarding local activation and distributed characteristics under different emotional states. Crucially, existing 2D evidence shows that high gamma power and network metrics achieve comparable classification accuracy through separate implementations. Their relative efficacy in VR remains unestablished. Considering the limitations of prior investigations, the present study primarily aims to (1) examine EEG differences in response to various emotions within VR environments to gain insights into the neural mechanisms underlying emotion processing, and (2) comparatively evaluate the emotion classification efficacy of two distinct feature types: high gamma band power and graph theory-based brain network topological metrics. EEG was recorded while subjects watched 4-s-long positive and negative VR video stimuli. Although various studies have used EEG for emotion recognition, most have employed 2D visual stimuli, and very few have examined EEG responses in VR environments. Given that the high-gamma band has been found to respond specifically to emotion in 2D environments (Li et al., 2019), we hypothesize that high gamma power will show valence-specific spatial patterns, with relatively higher gamma activity over temporal areas during negative states and higher gamma activity over prefrontal areas during positive states, as reported by Boucher et al. (2015). Additionally, we expect that high gamma features will yield superior classification performance compared to lower-frequency bands in this VR setting, consistent with Yang et al. (2020).

## 2 Materials and methods

### 2.1 Description of dataset

The study used the VREED (Yu et al., 2022), which recorded EEG signals from 19 healthy college students (13 males, 6 females; mean age 22.84, SD 1.50 years) from Shanghai University, all with normal or corrected-to-normal vision, when watching emotionally categorized (positive, neutral and negative) VR video clips, each lasting 4 s. All participants provided informed written consent, and the study was approved by the Shanghai Ethics Committee for Clinical Research, in compliance with the Declaration of Helsinki.

During the experiment, subjects were exposed to a series of VR stimuli (for detailed information, see Yu et al., 2022) designed to elicit positive, neutral and negative emotional responses. The VR videos were divided into two groups: one group contained 20 positive and 10 neutral videos, and the other group contained 20 negative and 10 neutral ones. To minimize emotional carryover effects due to the immersive nature of VR, the positive and negative videos were presented in distinct blocks. During the experiment, the order of presentation between video groups and within each video group was randomized using the stimulus presentation software. Participants viewed the stimuli while wearing HTC VIVE Pro VR headsets. It should be noted that although neutral video stimuli were included in the experiment, the paper that previously introduced the VREED dataset mainly analyzed the differences in EEG under positive and negative emotional stimuli (Yu et al., 2022). In this paper, we also focused on the high gamma band EEG characteristics under positive and negative stimuli. We have not yet analyzed the EEG characteristics under neutral stimuli. Future studies can compare the EEG differences between emotional stimuli and neutral stimuli.

**Figure 1:**
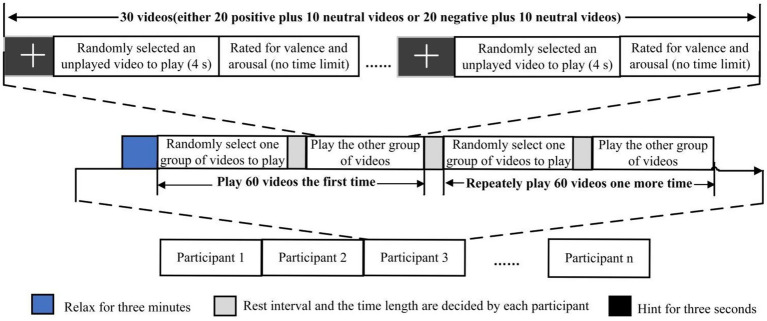
Protocol of emotion elicitation experiment (Yu et al., 2022).

As shown in Figure 1, the emotion induction protocol was conducted in two steps (adapted from Yu et al. (2022)). First, EEG data were acquired during the random presentation of the two video groups to each participant. Participants watched 30 videos, each preceded by a 3-s fixation cross (referred to as a “hint,” as shown in Figure 1) to help participants focus their attention. The data collected during these 3 s were used as the baseline. After each video, participants rated the emotional valence and arousal elicited by the video. Arousal and valence were assessed using a 9-point Likert scale, with 1 indicating calm/sadness and 9 indicating happiness/excitement. A short pause followed each group. The two video groups were played twice, and each participant underwent a total of 120 EEG trials (see Figure 1).

Participants were instructed to remain seated comfortably against the chair back, avoid excessive movements or facial expressions, and keep their gaze fixed during the experiment. These measures were taken to minimize electromyographic contamination of the EEG, which overlap with the high-gamma range. EEG signals were recorded using a 64-channel wireless EEG system (Neuracle Technology, Changzhou, China) at a sampling rate of 1,000 Hz, following the 10–10 International system (Yu et al., 2022). Impedances were maintained below 5 kΩ. FCz served as the recording reference, and Afz as ground. Due to VR headset constraints, dedicated vertical and horizontal EOG electrodes were not applied. However, frontal channels (e.g., FP1/2, FPz, AF7/8, F7/8) also provided sensitivity to ocular activity and can be used to remove ocular activity in the following preprocessing steps.

**Figure 2:**
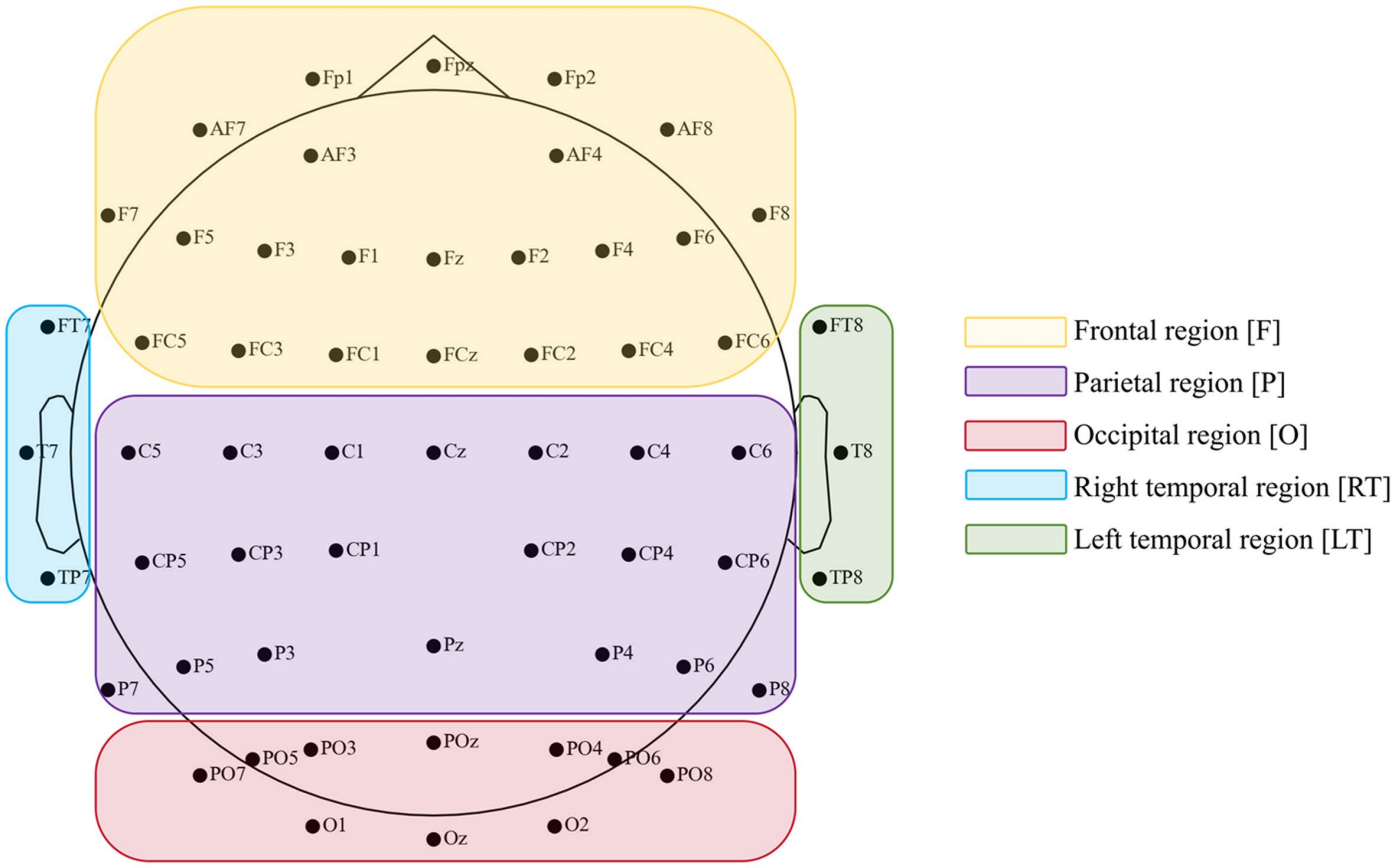
Position of 59 data electrodes based on an extended International 10–20 system (10–10 system).

EEG data were pre-processed offline using the EEGLab toolbox (Delorme and Makeig, 2004). From the original 64 electrodes, we first removed 5 unused channels (one cardiac electrode and 4 electrooculography electrodes), leaving 59 electrodes covering five brain regions (frontal, central-parietal, occipital, right temporal, and left temporal) as shown in Figure 2. Continuous EEG data were then band-pass filtered between 2 and 80 Hz and notch-filtered at 50 Hz and its harmonic at 100 Hz. Bad channels with persistent noise or poor connection were interpolated using spherical interpolation. Epochs from −1,000 ms to 4,000 ms relative to stimulus onset were extracted. Trials with visible large-amplitude artifacts were manually rejected. To enhance signal quality, especially in the high-gamma band, we implemented independent component analysis (ICA) on the remaining data. Components reflecting muscle activity, eye blinks, or other non-neural artifacts were identified based on their scalp topography, time courses, and power spectra. This approach is supported by prior literature showing that reliable high-gamma information can be extracted from scalp EEG when comprehensive artifact removal is applied (Gopan and Sinha, 2023). Finally, we rejected any trial with absolute amplitudes exceeding ±100 μV after artifact removal and re-referenced to an average reference using the REST algorithm (Yao, 2001).

### 2.2 EEG feature extraction

In previous studies, feature extraction has played a crucial role in emotion recognition systems, as the extracted features provide valuable information that enhances the predictive power of machine learning models. The primary goal of feature extraction is to identify the most concise and informative representation of EEG data, enabling an effective mapping of EEG signals to emotional states. In our study, as an initial step towards effective EEG-based emotion recognition, we extracted spectral power and functional brain network topological properties from the high gamma band (53-80 Hz) of EEG signals. We used these features to identify those most capable of distinguishing emotional states.

#### 2.2.1 Spectral power features

According to previous work (Yu et al., 2022), we found that the power spectral density (PSD) is efficient in characterizing local brain activations. The PSD is a stationary signal processing method suitable for processing narrowband signals. In our current study, we extracted the absolute power of the high gamma band using Welch’s average periodogram method, which was subsequently utilized as classification features. The computational procedures are as follows:

Given a time series *x*, we first divided it into *L* overlapping segments, each containing *K* data samples. To mitigate spectral leakage and improve frequency resolution, we implemented a 50% overlap between consecutive segments. Here, *K* equals 500, which is derived from the product of the window size (0.5 s) and the sampling rate (1,000 Hz). For each data segment, the periodogram was performed, defined as the squared magnitude of the Fourier transform of the segment, normalized by the segment’s length. For the *mth* segment *x_m_(n)*, the formula for the periodogram is given as follows (Equation 1):


(1) $${\mathrm{PSD}}_{m}(f)=\frac{1}{K}|\sum_{n=0}^{K-1}x_{m}(n)w(n)e^{-j2\mathrm{\pi fn}}|2$$

where w(n) is a window function to decrease spectral leakage effects, j is the imaginary unit, and K is the number of samples in each segment.

After calculating the periodograms for all segments, we averaged these estimates to derive the final PSD estimate. The Welch estimate of the PSD is expressed as follows (Equation 2):


(2) $$\mathrm{PSD}=\frac{1}{L}\sum_{i=0}^{L-1}{\mathrm{PSD}}_{i}(f)$$

Where L denotes the total number of segments, and ${\mathrm{PSD}}_{i}(f)$ represents the periodogram of the *ith* segment at frequency f .

#### 2.2.2 Functional brain network features

Most previous research studies focusing on the interactive activities of brain areas involved in affective perception agree that complex brain behaviors, such as emotion processing, are underpinned by the interaction among various brain regions (Yang et al., 2020; Zhou et al., 2023). In particular, emotional activities are associated with the dynamic organization of brain sub-networks; for example, high arousal is related to increased brain activity in the visual and dorsal attention networks (Nummenmaa et al., 2012). In this context, studies have shown that functional connectivity (FC) can reflect interactions between brain areas. Among the various methods for analyzing FC, phase synchronization is particularly noteworthy. It measures the correlation between brain areas based on the theories of “Binding by Synchrony” and “Communication through Coherence.” This approach has been shown to be effective for emotion recognition and for studying emotional mechanisms in large-scale network models (Li et al., 2019). In our current study, we constructed functional brain networks for the high gamma band in each trial using phase locking value (PLV) (Dasdemir et al., 2017), which is a reliable measurement of phase synchronization. First, 59 nodes within the network were defined, with each node corresponding to a channel on the scalp. The construction of the brain networks proceeded as follows: (1) estimating the functional connectivity using PLV between all possible pairs of channels, resulting in a symmetric matrix of dimensions 59 by 59, and (2) constructing the brain networks at a specified connectivity density.

##### 2.2.2.1 Phase locking value (PLV)

As previously mentioned, PLV was employed as a measure of functional connectivity. Specifically, PLV quantifies the instantaneous phase difference between two signals within a specific narrowband frequency. It assesses the level of synchronization between two neural signals in a given frequency band and time window, indicating the extent to which they enter a phase-locked state, based on the following formula (Equation 3):


(3) $$\mathrm{PLV}=\frac{1}{N}|\sum_{t=1}^{N}e^{j(\theta_{1}(t)-\theta_{2}(t))}|$$

where N denotes the total number of sampling points; $\theta_{i}(t)\;$ represents the instantaneous phase value of the *ith* (*i* = {1,2}) time series at the time point t , defined as follows (Equation 4):


(4) $$\theta_{i}(t)=\arctan\frac{{\tilde{x}}_{i}(t)}{x_{i}(t)}$$

where ${\tilde{x}}_{i}(t)$ refers the Hilbert transform of time series $x_{i}(t)$ corresponding to an EEG time series of a specific band. The Hilbert transform of a discrete-time signal x(t) could be directly computed according to the Equation 5 (Luo et al., 2009; Virtanen et al., 2020).


(5) $$x_{a}(t)=F^{-1}(F(\;x(t))2U)=x(t)+\tilde{x}(t)$$

where *j* is the imaginary unit, *U* is the unit step function, *F* refers to the Fourier transform, $F^{-1}$ denotes the inverse Fourier transform, and $\tilde{x}(t)\;$ represents the Hilbert transform of x(t) . PLV ranges from 0 to 1, with higher values indicating stronger synchronization between the calculated pairs and lower values representing weaker connectivity. By calculating the PLV values between all pairs of electrodes, we obtained a 59 × 59 adjacency matrix for the gamma frequency band, which was subsequently used for network analyses.

##### 2.2.2.2 Constructing brain networks

Subsequent to the computation of connectivity matrices, we employed graph theory to explore the organization of network patterns and to characterize meaningful functional segregation and integration of the human brain. The brain network is mapped by a set of vertices represented by brain regions, and edges that identify the connectivity patterns between different brain areas. For the construction of brain networks, we applied a threshold T to adjacent matrices, where the connectivity measure is set to 1 if it exceeds the threshold, and set to 0 in the opposite case. The obtained undirected binary network is used to calculate the network’s topological metrics in terms of network connection density, nodal, global and local efficiencies. Specifically, the network connection density refers to the number of edges in the network presented as a proportion of actual connections relative to the total possible connections within the network (Equation 6).


(6) $$p=\frac{2E}{\left(N^{2}-N\right)}$$

where *N* and *E* refer to the number of nodes and edges, respectively. In this paper, the topological properties were computed at a connectivity density of 14%.

Nodal efficiency is a measure of integration that reflects the ability of a node to transmit information to other nodes in a network. It provides insights into the significance of a given node in maintaining efficient communication within the brain network. For node j, nodal efficiency is calculated as the normalized sum of the reciprocals of the shortest path lengths from *j* to all other nodes (Equation 7):


(7) $$E_{\text{nodal}}(j)=\frac{1}{N-1}\sum_{\begin{array}{c} j,k\in V\\ j\neq k \end{array}}\frac{1}{d_{\mathrm{jk}}}$$

where *V* represents all the nodes in the network, *d_j,k_* denotes the number of steps of the shortest path between nodes *j* and *k*.

The global efficiency quantifies the overall efficiency of information transfer within a brain network and is determined as the average inverse of the shortest path length between all nodes in the network according to Equation 8:


(8) $$E_{\text{global}}(G)=\frac{1}{N}\sum_{j\in V}E_{\text{nodal}}(j)$$

where *E_nodal_(j)* refers to the nodal efficiency of network node *j*. Global efficiency is a scaled measure varying from 0 to 1, with a value of 1 reflecting maximum global efficiency in the network.

Regarding local efficiency, it serves as a metric for evaluating brain functional segregation, reflecting the efficiency of information transfer within a specific brain network. It provides insights into the efficacy of information transfer among the neighbors of a specific node when the node itself is eliminated. The local efficiency of node *i* is defined as the efficiency of information transfer within the subgraph *G_i_*, which is composed of *i*’s neighboring nodes, encompassing all nodes directly connected to *i* and the edges between those neighboring nodes. For node *i*, its local efficiency *E_local_ (i)* is defined as Equation 9:


(9) $$E_{\text{local}}=\frac{1}{N_{G_{i}}(N_{G_{i}}-1)}\sum_{\begin{array}{c} j,k\in N_{G_{i}}\\ j\neq k \end{array}}\frac{1}{d_{\mathrm{jk}}}$$

Where $d_{\mathrm{jk}}\;$ represents the shortest path distance between node *j* and *k* within subgraph *G_i_*. High local efficiency indicates a topological organization that reflects a resilient and robust local network structure surrounding a particular node. Conversely, low local efficiency signifies a poorly connected local network structure that is more vulnerable to disruption.

### 2.3 Statistical tests

In this paper, an ANOVA was used to statistically analyze the power spectrum and the network topology characteristics of scalp EEG in the high gamma band (53–80 Hz) during virtual reality video stimulation with different emotional attributes.

For the power spectrum analysis, a two-way repeated measures ANOVA with two within-subject factors, Region (Frontal [F], Central-parietal [P], Occipital [O], Left-temporal [LT], Right-temporal [RT]) and Emotion (Negative [Neg], Positive [Pos]), was conducted to assess the effects of emotion type on the power across different brain regions. Likewise, a two-way repeated measures ANOVA with Hemisphere (Left-frontal [LF]: Fp1, AF3 and AF7; Right-frontal [RF]: Fp2, AF4 and AF8) and Emotion (Negative, Positive) as within-subject factors was applied to examine the asymmetry in prefrontal power between the two hemispheres in the high gamma frequency band. Regarding network features, differences in global and local efficiencies between positive and negative emotions were evaluated using a paired *t*-test.

Upon identifying a significant interaction effect in the two-way repeated measures ANOVA, a simple effect analysis was carried out to further explore the nature of the interaction. Post-hoc pairwise comparisons were conducted and corrected using the Bonferroni method to account for multiple comparisons. All statistical analyses were conducted using SPSS version 20.0 (SPSS Inc., Chicago, IL). Any violations of sphericity were addressed using the Greenhouse–Geisser epsilon correction. Results were considered statistically significant at *p* < 0.05 with uncorrected degrees of freedom and the corrected *p*-values reported.

### 2.4 Emotional state classification

#### 2.4.1 Classification analysis

While traditional statistical methods are known to be more suitable for hypothesis testing in the analysis of individual EEG features, machine learning methods are more effective for exploring complex, high-dimensional relationships among EEG features. In this study, we also used a support vector machine (SVM) to perform classification analysis on the extracted EEG features (Wang et al., 2020). In this study, the SVM uses a radial basis function (RBF) kernel to map input data to a higher-dimensional space, improving class separability and enabling more effective classification.

**Figure 3:**
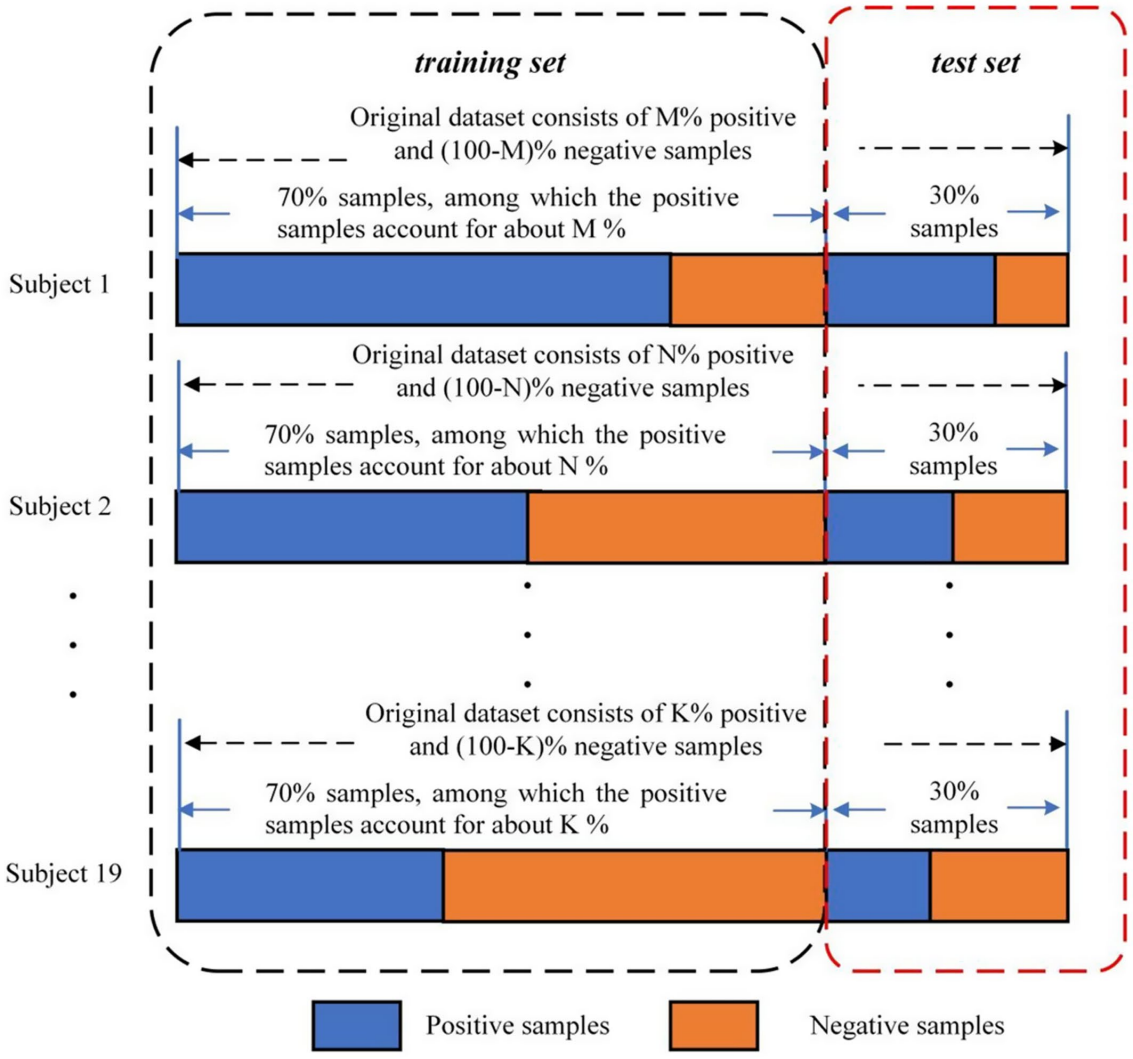
Division of the VREED dataset used for training and test (Yu et al., 2022).

For our analysis, we treated each EEG trial as an independent sample and performed a binary classification task to distinguish between positive and negative emotional states using EEG power and brain network features. First, the data for each participant was divided into a 70% training set and a 30% test set. Stratified sampling was used to ensure that the proportion of each emotion class in both the training and test sets remained consistent with the original dataset (see Figure 3, adapted from Yu et al. (2022)). Then, the SVM classifier was trained on the combined training data from all participants. Finally, to evaluate the model’s performance, the test set was used to calculate the classification accuracy. To mitigate the influence of random splits on the classification results, we repeated the train-test process 10 times, and the average classification accuracy across these 10 iterations was used as the final performance measure, providing a more stable and reliable evaluation metric.

#### 2.4.2 Feature importance analysis

To improve the interpretability of the classification model and identify key neural signatures, we performed feature selection following the initial binary classification analysis. Specifically, features that demonstrated the highest classification accuracy during the initial analysis were further evaluated using a random forest model, implemented with the Python package scikit-learn. The aim was to determine the importance of these features at the regional level, identifying which electrodes or brain regions contributed most significantly to the classification outcome.

The feature importance analysis was conducted using an impurity-based metric, the Gini importance (or mean decrease impurity), which measures each feature’s contribution to reducing classification uncertainty. The approach computes impurity-based feature importance, specifically using Gini importance (or mean decrease impurity) to evaluate the significance of each feature in the classification process. In detail, the Gini importance of a feature within a single decision tree is determined by calculating the reduction in the Gini index (from parent nodes to child nodes) that is brought by the given feature. For the random forest model, the overall Gini importance of a feature is obtained by averaging its Gini importance across all the trees in the forest. Features that exhibit higher Gini importance are considered more influential in contributing to the classification outcome. This feature importance analysis is valuable for revealing the discriminative potential inherent in regional physiological signals, thereby enhancing classification performance, reducing computational complexity, and providing potential insights into the neural mechanisms underlying different emotional states.

## 3 Results

### 3.1 Power spectral analysis

**Figure 4:**
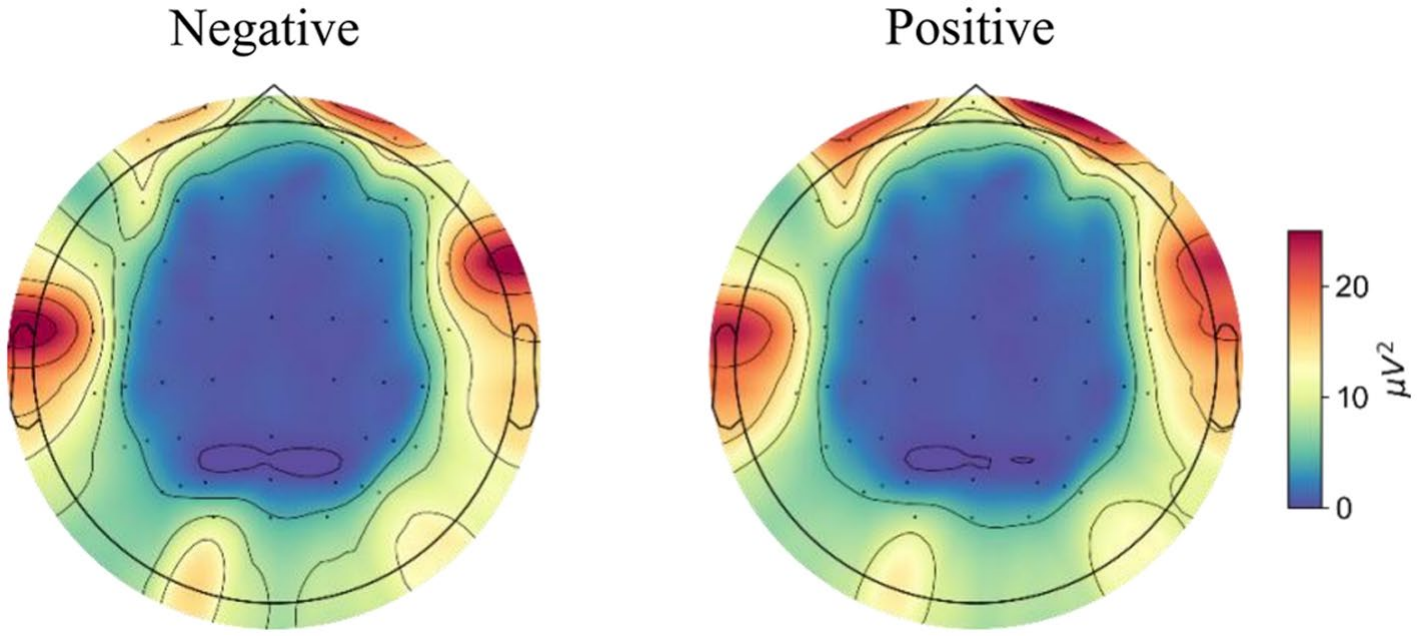
The topological maps of averaged power across trials for negative and positive emotional states.

Figure 4 shows the topographic maps of the averaged PSD across trials for both negative and positive emotional states. Specifically, higher gamma band power was quantified in the prefrontal regions during the positive emotional state compared to the negative state. The power spectra of different brain regions and hemispheres under positive and negative emotions were statistically assessed using a two-way repeated measures ANOVA and the results were reported in the following section.

#### 3.1.1 Regional spectral analysis

The effects of emotional type on the power of brain regions were evaluated using a two-factor repeated-measures ANOVA with two within-subject factors of Region (F, P, O, LT, RT) and Emotion (Neg, Pos). The analysis revealed a significant main effect of Region [*F*(4,72) = 10.346, *p* < 0.001, $\eta_{p}^{2}$ = 0.365], as well as a significant interaction effect of Emotion*Region [*F*(4,72) = 5.273, *p* < 0.001, $\eta_{p}^{2}\;$ = 0.227].

**Figure 5:**
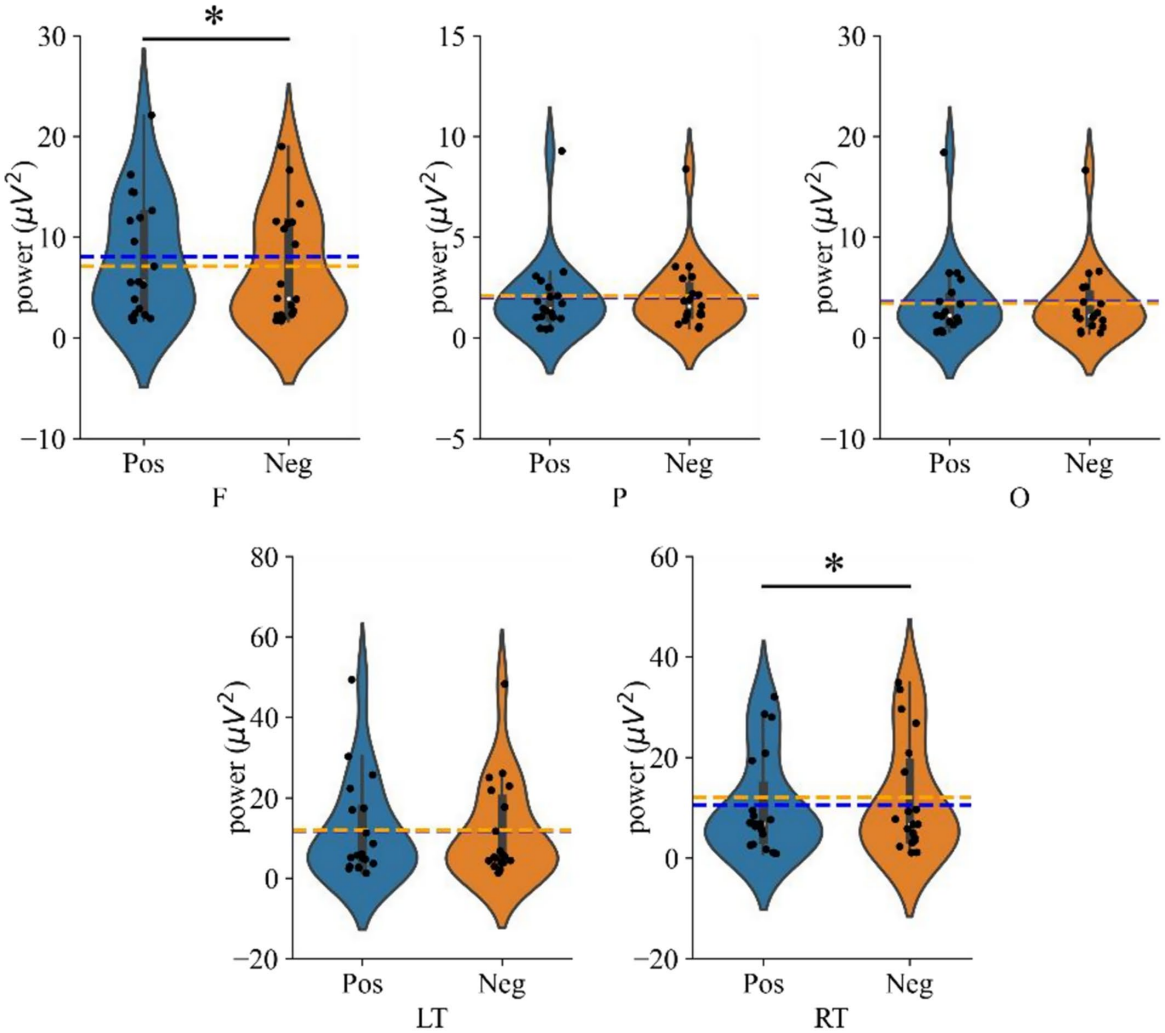
Violin plots showing average power across brain regions under different emotional states (Positive vs. Negative). The dotted line indicates the average value; where ‘F’, ‘P’, ‘O’, ‘LT’, ‘RT’ denote frontal, parietal, occipital, left temporal and right temporal regions, respectively, and ‘*’ denotes *p* < 0.05.

Subsequent simple effects analysis showed that the mean power in the frontal region was significantly higher during the positive emotional state compared to the negative state (*p* = 0.020, Cohen’s *d* = 0.171). Conversely, in the right temporal region, the mean power was significantly higher during the negative emotional state compared to the positive one (*p* = 0.037, Cohen’s *d* = 0.139). Figure 5 illustrates these comparisons of average power in each brain region under different emotional states, with significant effects indicated by * (*p* < 0.05).

#### 3.1.2 Frontal asymmetry

**Figure 6:**
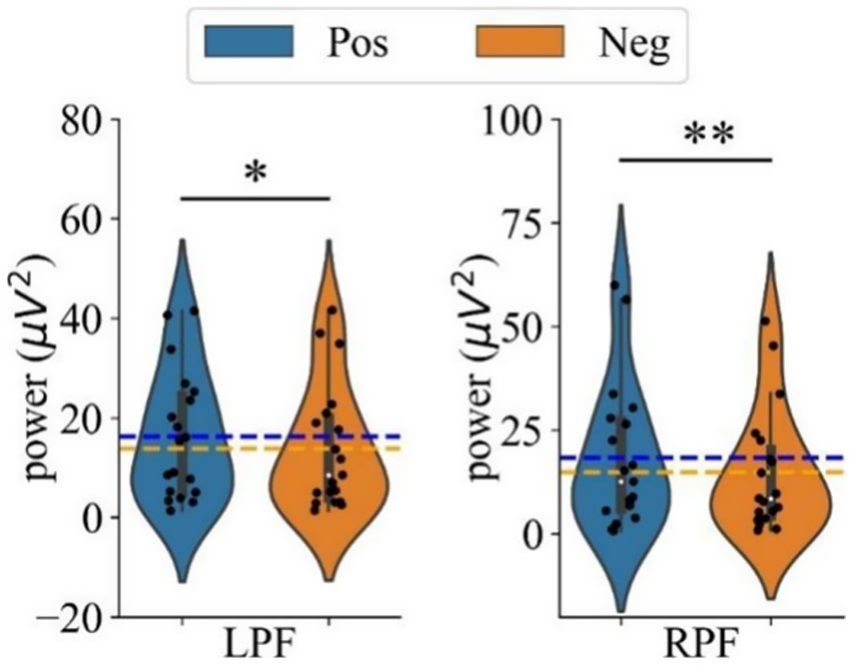
The mean power in the gamma band during positive and negative emotional states for the left prefrontal (LPF, left panel) and right prefrontal (RPF, right panel) regions. Difference in power between positive and negative emotional states. ‘*’ Indicates *p* < 0.05 and ‘**’ indicates *p* < 0.01.

The effects of different emotional stimuli on left and right prefrontal power in the gamma band were analyzed using a two-way repeated measures ANOVA with two within-subject factors of Hemisphere (LF, RF) and Emotion (Neg, Pos). As reported in Figure 6, two significant effects were found: a main effect of Emotion [*F*(1,18) = 9.087, *p* = 0.007, $\eta_{p}^{2}\;$ = 0.335] and an interaction effect of Emotion*Hemisphere [*F*(1,18) = 4.767, *p* = 0.043, $\eta_{p}^{2}\;$ = 0.209].

To further explore the interaction, simple effects analyses were conducted. These analyses revealed that the power in both the left (*p* = 0.013, Cohen’s *d* = 0.188) and right prefrontal regions (*p* = 0.006, Cohen’s *d* = 0.213) was significantly greater with positive stimuli compared to negative stimuli. Figure 6 shows the comparison of average gamma band power in the left and right prefrontal regions under positive and negative emotional states, with power consistently higher under positive stimuli for both hemispheres.

### 3.2 Brain network analysis

**Figure 7:**
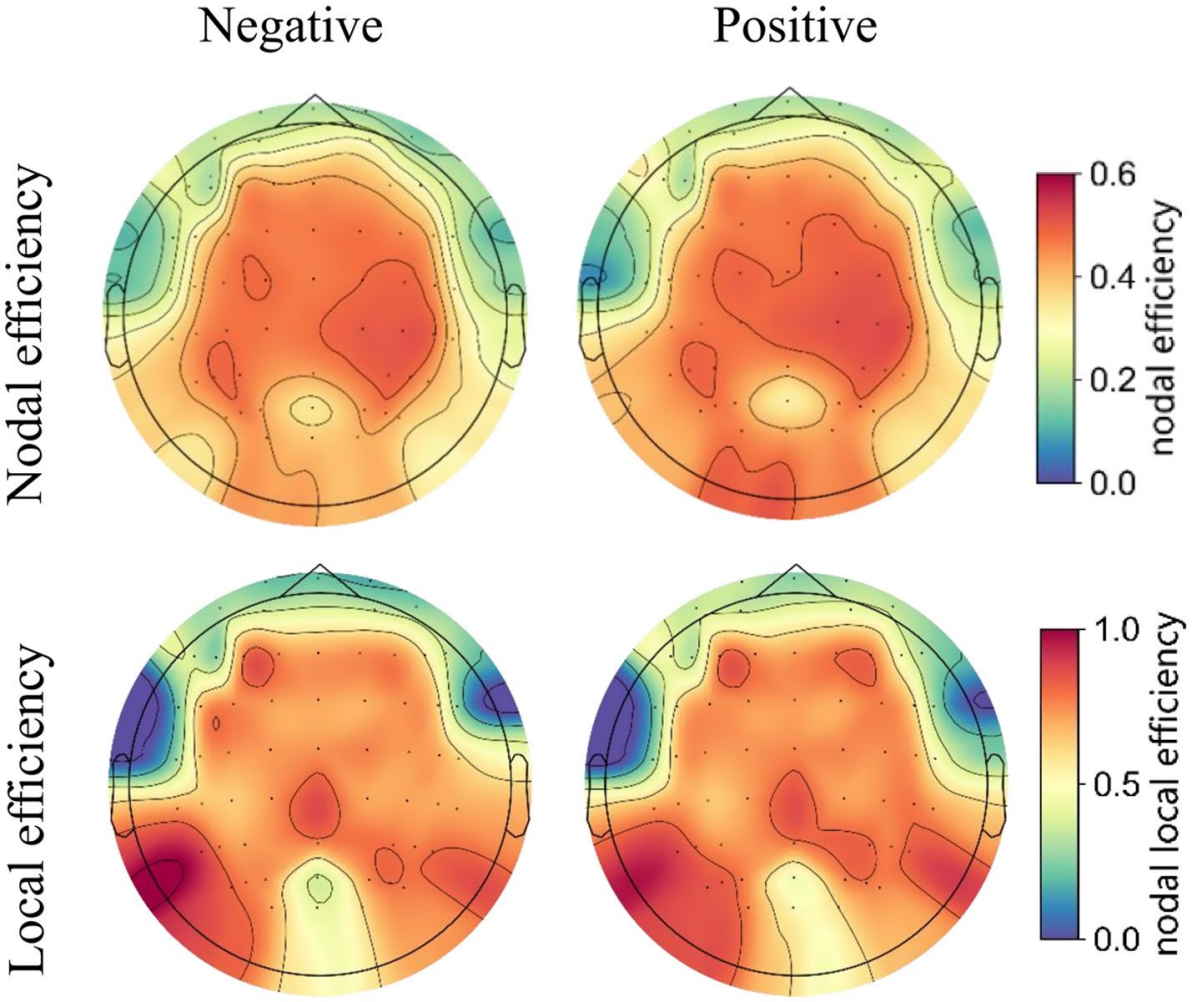
The topographic maps of the grand averaged nodal efficiency **(upper panel)** and local efficiency **(lower panel)** of the high gamma-band EEG brain network under positive and negative emotional states.

Functional connectivity matrices were constructed using the PLV method, and brain networks were extracted at a connectivity density of 14%. The topographic maps of the grand averaged nodal efficiency and local efficiency during negative and positive emotional states are shown in Figure 7, illustrating the spatial distribution of these network metrics.

The nodal efficiency was first averaged across the 59 nodes to obtain the global efficiency. A paired t-test revealed no significant difference in global efficiency between the negative and positive emotional states [*t*(18) = 1.241, *p* = 0.230]. This indicates that the overall brain network integration remains similar across both emotional states.

**Figure 8:**
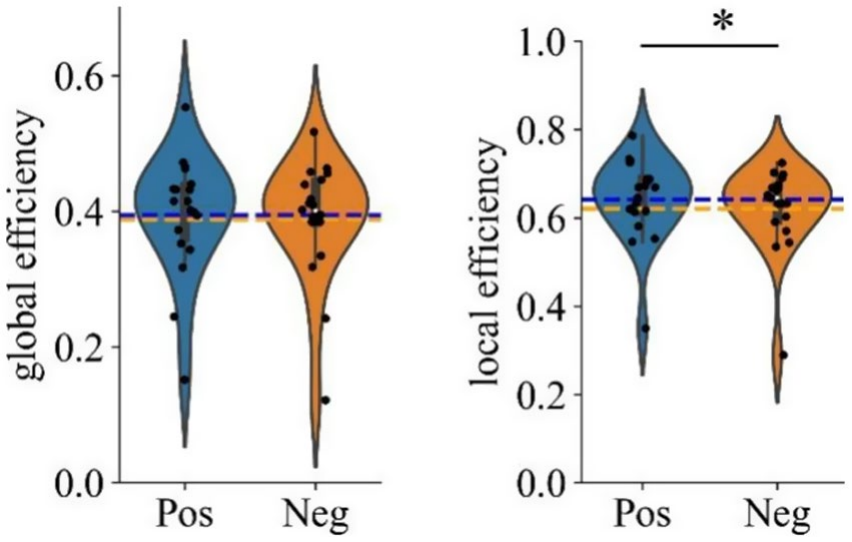
Violin plots showing grand averaged global and local efficiency under different emotional states (Positive vs. Negative). ‘*’ Indicates *p* < 0.05.

Considering the absence of significant differences in global efficiency, we proceeded to examine local efficiency to identify potential differences in the localized organization of brain networks between the two emotional states. The average local efficiency across the 59 electrodes was first computed, followed by a paired *t*-test, which revealed significantly higher values for positive emotional states compared to negative emotional states in the gamma band [*t*(18) = 2.792, *p* = 0.012 (pos > neg)]. This finding suggests that brain networks under positive emotional states are organized in a more modular way, which enhances local information processing and may contribute to greater resilience against network perturbations. Figure 8 shows the statistical results for both global and local efficiency during negative and positive emotional states, with ‘*’ indicating a significant effect (*p* < 0.05).

### 3.3 Emotional state classification results

#### 3.3.1 Classification results

**Figure 9:**
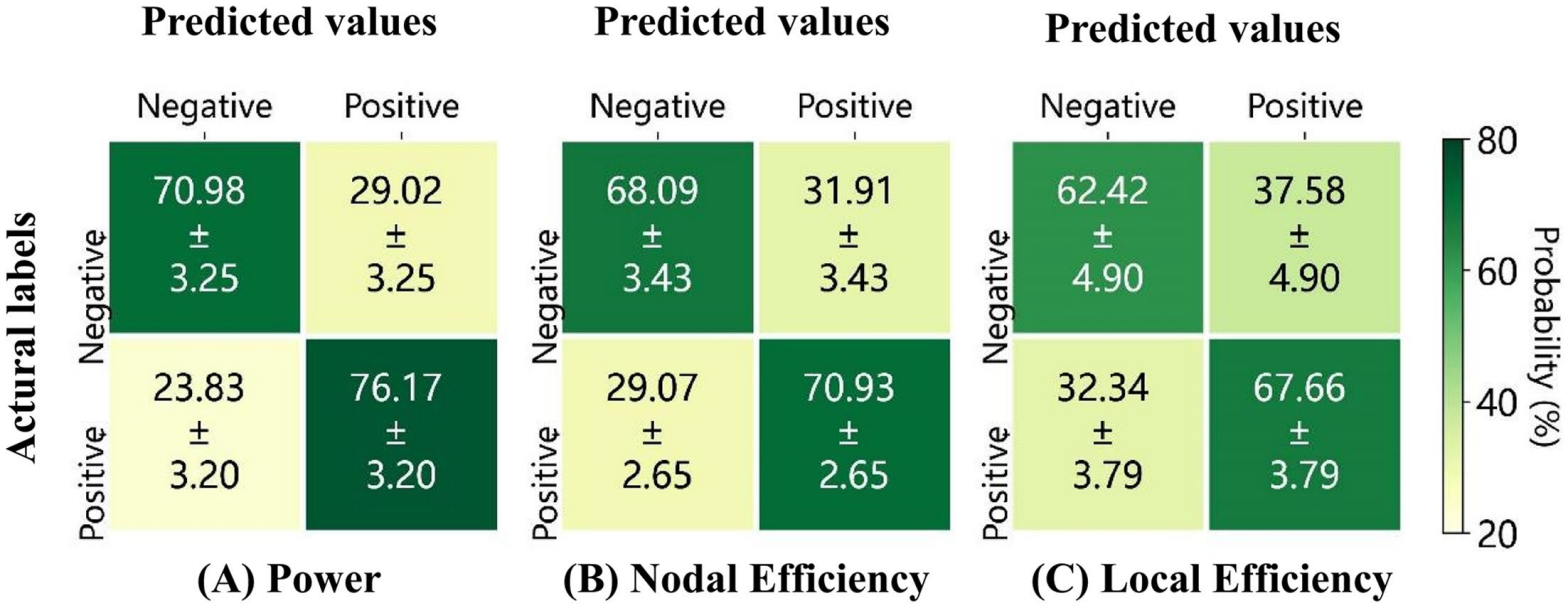
Average confusion matrices for 10 repetitions of classification based on **(A)** the power, **(B)** the nodal efficiency, and **(C)** the local efficiency, derived from the high gamma band EEG of 59 electrodes.

Figure 9 presents the average confusion matrices for the classification of positive and negative emotional states based on three feature sets: (A) high gamma band power, (B) nodal efficiency, and (C) local efficiency derived from 59 channels. The highest overall classification accuracy was observed for high gamma band power features, reaching 73.57 (2.30), followed by nodal efficiency at 69.51 (2.62), and local efficiency at 65.03 (1.33).

**Figure 10:**
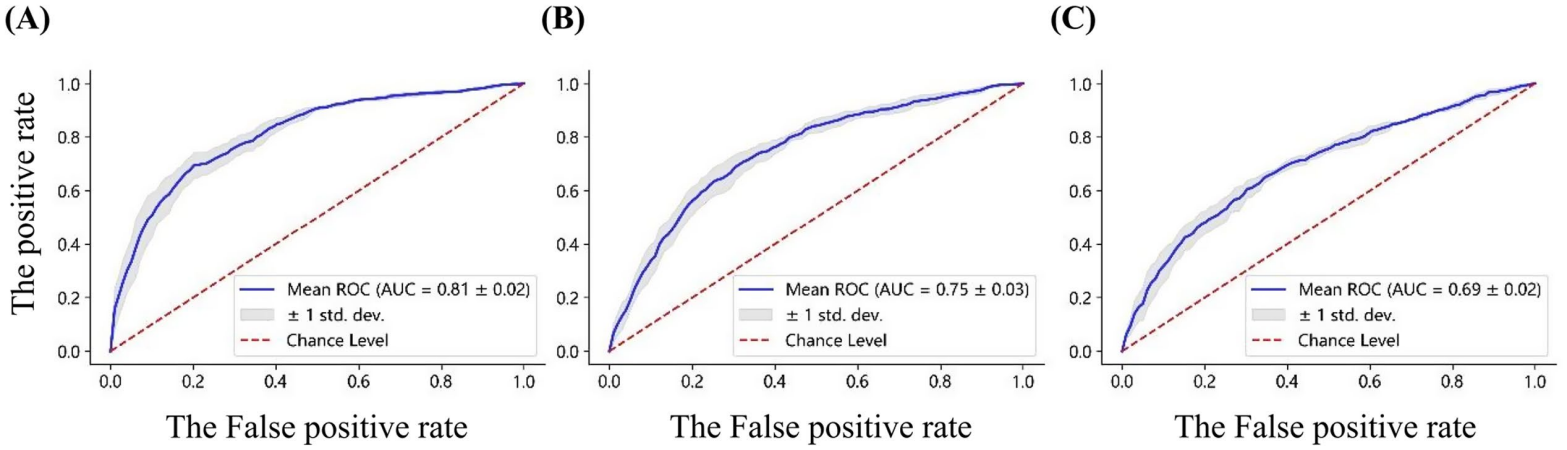
ROC curves for classification based on **(A)** the power, **(B)** nodal efficiency, and **(C)** local efficiency, derived from the high gamma band EEG of 59 electrodes. The AUC values are reported for each feature set.

To further evaluate the discriminative performance of these feature sets, Receiver Operating Characteristic (ROC) curves were analyzed, as shown in Figure 10. High gamma band power achieved the highest classification performance, with an AUC of 0.81 ± 0.02, followed by nodal efficiency (AUC: 0.75 ± 0.03), and local efficiency (AUC: 0.69 ± 0.02). These ROC results align with the classification accuracies indicated in the confusion matrices, reinforcing that high gamma band power features derived from 59 electrodes provide the most effective features for differentiating emotional states. The classification methodology described above is consistent with the approach originally employed in the development of the VREED dataset. In that work, an SVM classifier was systematically used to independently evaluate the discriminative power of both functional network topological characteristics and spectral power density features across four conventional frequency bands: theta, alpha, beta, and gamma. To ensure methodological continuity and enable robust cross-frequency comparisons, we adopted this same analytical framework in the present study to evaluate neural signatures in the high gamma band, thereby allowing for direct benchmarking against previously established results from lower frequency bands.

#### 3.3.2 Feature importance analysis results

Given that high gamma band power has the highest classification accuracy, further analysis was conducted to determine which regions or channels’ high gamma band power were most critical in differentiating EEG patterns between positive and negative emotional states. Feature selection was employed to identify the most relevant features, aiming to enhance the interpretability of the classification model. This analysis was performed using a Random Forest model, with the following steps:

(1) Dataset division: the dataset was divided into training and test sets in a ratio of 7:3.
(2) Model training: a Random Forest model was trained on the training set, and the Gini importance of each feature was calculated, indicating the importance of each feature in the classification process.
(3) Feature ranking and validation: features were ranked in descending order based on Gini importance, and subsets of features (the top n features) were sequentially selected to evaluate their classification performance on the training set.
(4) Repeatability validation: The above steps were repeated 5 times to obtain the average classification accuracy and Gini importance values.

**Figure 11:**
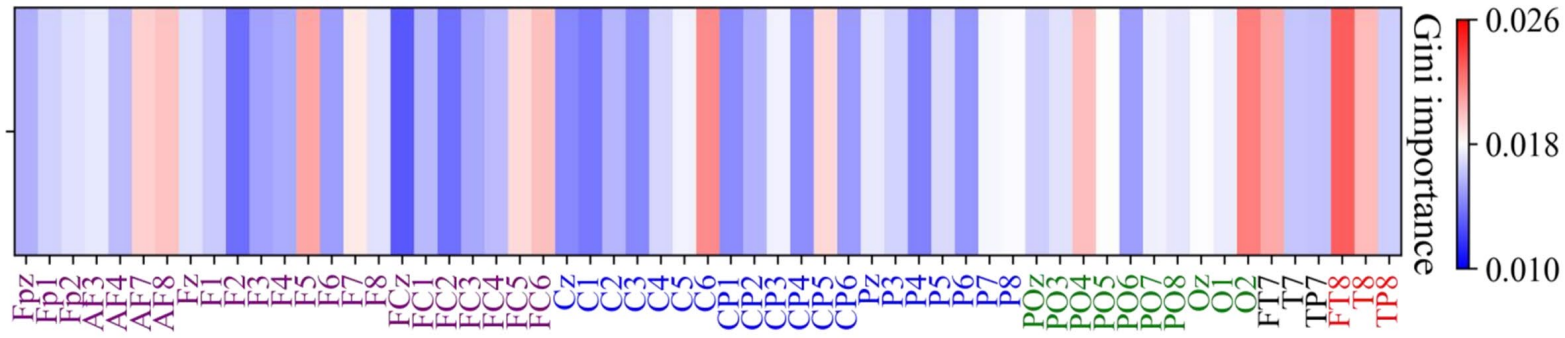
Channel-wise feature importance heatmap using Gini importance values. The Gini importance values were computed from Random Forest analysis of high gamma band power features, indicating the contribution of each channel to the classification between positive and negative emotional states. Larger Gini importance values denote feature that are more relevant in the classification process.

Figure 11 illustrates the Gini importance for each feature calculated by the Random Forest model. It can be observed that the highest Gini importance values are concentrated in the temporal (FT7, FT8 and T8) and occipital regions (O2), indicating a strong contribution to the classification task from these areas.

**Figure 12:**
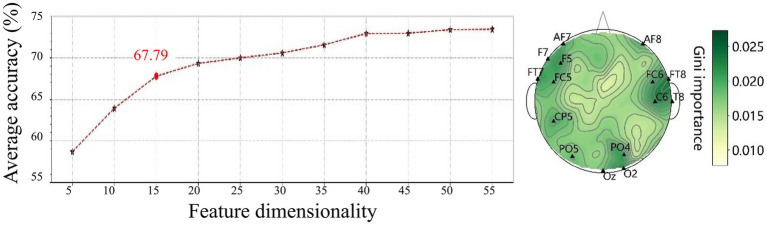
Classification accuracy **(left)** and spatial distribution of the top 15 features selected by Gini importance **(right)**. Most features are located in the frontal, temporal, and occipital regions, highlighting their significance in differentiating emotional states.

Subsequently, features were ranked by descending Gini importance values and their classification performance was evaluated based on subsets of increasing size (n ranges from 5 to 55 features, incremented by 5 each time). Figure 12 shows the classification accuracy for these subsets, revealing that the top 15 features achieved an accuracy of 67.79%. These selected features are primarily located in the frontal, temporal, and occipital regions, as shown in Figure 12, indicating the importance of these regions in the classification of emotional states.

## 4 Discussion

VR has increasingly become a valuable research tool in neuroscience, enabling the study of complex sensory and emotional experiences under immersive and controlled conditions (Valmorisco et al., 2024). However, the current understanding of neural responses to VR stimuli remains limited, and this gap may impact the accuracy and reliability of findings in fields such as emotion regulation and neurorehabilitation through neurofeedback training in a VR environment. To address these limitations, this study aims to characterize the neural characteristics of VR-induced emotional responses using scalp EEG, focusing on oscillatory brain activity and network connectivity patterns. Specifically, we analyzed local neural activity (regional high gamma band power and frontal asymmetry) and network-level functional integration (nodal efficiency and local efficiency) associated with positive and negative emotional responses induced by VR stimuli.

Our findings highlighted several key results: First, high gamma band power in the prefrontal region was significantly higher during positive emotional states compared to negative emotional states, while the right temporal region showed significantly greater high gamma band power during negative emotional states relative to positive emotional states. Second, emotional states had a significant effect on local brain network efficiency, with higher local efficiency observed during positive emotional states, indicating that brain networks might be more modular under positive emotional experiences, facilitating local information processing. Third, in terms of classifying emotional states, high gamma band power features exhibited the highest classification accuracy (73.57 ± 2.30%) compared to brain topological patterns. Feature selection further revealed that high gamma band power in the frontal, temporal and occipital regions played critical roles in distinguishing between positive and negative emotional states.

### 4.1 Neural signature of emotional states in the high gamma band

Through time-frequency analysis, we observed significantly higher gamma power in the prefrontal region during positive emotional states compared to negative states, while the right temporal region exhibited greater gamma power during negative emotions. These findings, showing higher frontal gamma for positive states and increased temporal gamma for negative states, align with our *a priori* hypotheses and are consistent with prior work by Boucher et al. (2015).

The increased high gamma activity in the prefrontal region during positive emotional states may reflect enhanced integration of multimodal sensory information with affective reactions (Li et al., 2019), positive affect maintenance and reward and approach-related behavior (Davidson, 2004). This is consistent with the prefrontal cortex’s (PFC) critical role in emotion experience and regulation through its top-down control on subcortical structure activity like the amygdala (Dixon et al., 2017). The prefrontal cortex, especially the dorsolateral and ventromedial part, is known to be involved in higher-order cognitive processes, including emotion regulation, which are particularly activated during positive emotional experiences (Davidson, 2004). A recent fMRI study showed that the activation of the vmPFC was significantly greater in response to pleasant stimuli compared to unpleasant ones, further emphasizing its particular role in processing positive emotion (Winker et al., 2018). Additionally, disruptions in vmPFC activity, as observed in depressive patients, have been connected to diminished positive emotion experiences, highlighting its importance in maintaining positive emotions (Alexander et al., 2023). In a recent study using functional near-infrared spectroscopy (fNIRS), researchers found that the right dlPFC showed significantly higher activation during positive emotions compared to negative emotions (Zhang et al., 2022). Enhanced prefrontal activation during positive emotions might indicate the increased cognitive control required to sustain a positive state, reflecting the role of this area in modulating positive emotional responses and approach behaviors.

On the other hand, the right temporal region exhibited significantly higher high-gamma power during negative emotional states. This finding is consistent with previous research linking the right temporal lobe to the perception and processing of negative stimuli (Sato and Aoki, 2006; Lukito et al., 2023), which supports the widely held view that negative emotions associated with withdrawal behaviors are preferentially processed by the right hemisphere (Davidson, 2004). Specifically, the increased high-gamma power observed in the right temporal region might reflect an evaluation and response to negative stimuli, which is crucial for adaptive behaviors involving threat detection and avoidance. Moreover, lesion studies have demonstrated the essential role of the right temporal lobe in recognizing fear expressions, further emphasizing its involvement in negative emotional processing (Midorikawa et al., 2019). A recent meta-analysis on brain network characteristics also revealed a significant link between the right temporal region and the processing of negative emotions (Arias et al., 2020). This evidence collectively supports the notion that the right temporal cortex plays a key role in processing negative emotions.

Our results did not find any significant lateralized activation in the prefrontal cortex during either positive or negative emotional states. This lack of significant lateralized activation in the prefrontal cortex during either positive or negative emotional states might be related to the characteristics of the high-gamma band itself. Previous research suggests that emotional lateralization effects are more commonly observed in the alpha band (8–12 Hz), which is more directly linked to cortical inhibition and activation balance (Li et al., 2021). The high gamma band, on the other hand, is typically associated with high-level cognitive processes (Kucewicz et al., 2024), integration of emotional information, and attentional focus (Ku et al., 2022), which may not exhibit the same degree of hemispheric asymmetry as alpha rhythms. This could partially explain why the current study did not observe significant prefrontal lateralization in the gamma band, and indicates that future studies interested in emotional lateralization should consider focusing on lower frequency bands, such as alpha.

### 4.2 Brain topological features

Recent research in neuroimaging and brain network analysis has revealed that emotions significantly influence the functional architecture of the brain by modulating functional brain network properties, such as connectivity and efficiency (Dan et al., 2023). In the current study, we found that positive emotional states significantly enhance local efficiency compared to negative emotional states, indicating a differential impact of emotional valence on brain network organization. Local efficiency is a measure of how efficiently information is exchanged within small brain subnetworks, reflecting the rapidity and effectiveness of communication among neighboring regions (Zhao et al., 2022). Higher local efficiency signifies improved intra-regional connectivity, which facilitates fast and effective information transfer, particularly within specialized regions involved in cognitive and emotional processing.

Our results are consistent with previous neuroimaging research suggesting that positive emotions can enhance the efficiency of functional brain networks (Liu et al., 2022). This alignment specifically corresponds to the broaden-and-build theory of positive emotions, which posits that positive emotions broaden an individual’s thought-action repertoire and consequently enhance cognitive flexibility and efficiency (Fredrickson, 2001). The observed enhancement in local efficiency during positive emotional states may reflect enhanced intra-regional communication that facilitates flexible and efficient cognitive processing (Franceschini et al., 2022). Therefore, the utilization of VR technology to induce positive emotional states holds significant potential for enhancing local brain network efficiency. This approach can be leveraged to develop more effective learning, therapeutic and entertainment applications.

### 4.3 Classification results

The results of emotional state classification indicate that spectral power density features in the high gamma band achieved an accuracy of 73.57% ± 2.3% in distinguishing EEG patterns associated with positive and negative emotional states. This performance exceeds the classification accuracies reported in previous studies (Yu et al., 2022) for theta (71.35% ± 2.01%), alpha (65.56% ± 2.13%), beta (63.14% ± 1.90%), and low-gamma (68.30% ± 2.01%) bands. These findings are consistent with those reported by Yang et al. (2022), who used 2D emotional stimuli and found that high gamma band EEG signals are more sensitive and effective than other frequency bands in studying human affective perception. Additionally, the classification accuracies based on functional network features in the high gamma band were 69.51% ± 2.62% for nodal efficiency and 65.03% ± 1.33% for local efficiency, both of which were lower than the accuracy achieved using spectral power density features (73.57% ± 2.3%). Although network-based features provide valuable insights into global and inter-regional connectivity patterns, the present results suggest that localized neural activity, reflected in gamma power, carries more direct and discriminative information for classifying emotional states. Gamma oscillations, often linked to neural synchronization and the integration of distributed brain networks, have been shown to play a central role in emotion processing and attentional resource allocation (Kaiser and Lutzenberger, 2003; Colgin and Moser, 2010). According to the bottleneck theory, attention is not an unlimited resource and must be efficiently distributed across different sensory modalities (Wahn and König, 2015). This distribution becomes particularly critical in VR environments, where users must process both visual and spatial information simultaneously.

Notably, the top 15 features, predominantly localized in the frontal (AF7, AF8, F5, F7, FC5, FC6), temporal (FT7, FT8, T8), and occipital regions (PO4, PO5, Oz, O2), resulted in a classification accuracy of 67.79%. These findings also underscore the critical role of localized spectral power in the classification of emotional states, while highlighting the spatial and spectral specificity of emotion-related neural activity. The top 15 features achieved a classification accuracy of 67.79%, suggesting that a compact feature set can effectively balance classification accuracy and computational efficiency. This is particularly important for real-time emotion recognition systems, where minimizing computational demands is essential for ensuring prompt responses.

Among the features selected with the random forest based on impurity-based feature importance, it was evident that electrodes located in the frontal, temporal and occipital regions played a pivotal role in emotion recognition within the VR environment. As mentioned by previous studies, the temporal cortex is particularly relevant due to its involvement in auditory and social–emotional processing, including the recognition of facial expressions (Sliwinska and Pitcher, 2018). The occipital cortex, is essential for visual emotional processing, particularly in response to emotionally salient visual stimuli (Lang et al., 1998). The frontal cortex is fundamental to the generation, perception and regulation of emotions (Ochsner et al., 2012; Huang et al., 2021). Previous research indicated that the VR intervention in the high gamma band, triggered paths between, the occipital and prefrontal areas, creating a kind of synergy that reflects the dynamic allocation of attentional resources from the occipital lobe’s visual response to the prefrontal lobe’s decision-making and the motion control of the neural motor regions, while completing VR tasks (Yang et al., 2022). This regional specificity of high gamma power aligns well with previous literature, reinforcing the idea that emotional processing relies on a distributed network involving both sensory and higher-order evaluative regions.

This study investigated the neural dynamics of emotion processing in VR by analyzing high gamma spectral power and functional brain network organization. Three main findings were presented: First, power spectral analysis revealed valence-specific spatial patterns of neural activity. Gamma power was significantly higher in the frontal cortex during positive emotional states, while negative emotional states correlated with increased right temporal gamma power. These findings demonstrate neural differentiation of emotional valence and suggest that gamma oscillations in specific brain regions modulate emotional experiences. Second, while graph-theoretic metrics capture distributed network dynamics, spectral power features exhibited superior classification accuracy (73.57% vs. 69.51%), emphasizing their practical value for real-time emotion recognition systems. Finally, feature importance analysis underscored the significance of frontal, temporal, and occipital regions in emotion processing in VR, indicating engagement of both evaluative and sensory neural networks.

These results not only support the role of high gamma band power in emotion processing but also extend our understanding by demonstrating that these biomarkers can effectively classify emotional responses in immersive VR virtual environments. This contributes to the growing evidence of the utility of high gamma oscillations as an indicator for emotional differentiation in both naturalistic and controlled settings.

The present study has several limitations, including a restricted sample size (*N* = 19) and binary emotion classification. Future research should also expand methodological approaches by incorporating neutral states, implementing longitudinal designs, and integrating multimodal neuroimaging techniques such as functional near-infrared spectroscopy and eye-tracking to enhance feature interpretability. These findings establish a neurocomputational framework for optimizing emotion recognition in immersive environments, with potential applications in adaptive virtual reality therapies, neurofeedback training, and affective brain-computer interfaces.

## Data Availability

The dataset was obtained from Yu et al. (2022) with written permission from the corresponding author (Dr. Yingjie Li). The dataset is not directly available in a public repository. While the corresponding of this article (Dr. Minchang Yu) is the first author of Yu et al. (2022), data ownership remains with their original corresponding author (Dr. Yingjie Li). Access requests should be directed to Dr. Yingjie Li.
